# The geometry of Nature’s stingers is universal due to stochastic mechanical wear

**DOI:** 10.1073/pnas.2526098123

**Published:** 2026-03-06

**Authors:** John Sebastian, Kaare H. Jensen

**Affiliations:** ^a^Department of Physics, Technical University of Denmark, Kongens Lyngby DK-2800, Denmark

**Keywords:** biostingers, morphological convergence, mechanical wear, erosive processes, geometric flow

## Abstract

Despite their ubiquity in Nature, spikes or stingers rarely exhibit sharp tips. Instead, a closer inspection of their roughly conical tips reveals a striking similarity in their profiles: They adhere to a power-law, $z∼r^{n}$, where n≈2. This conformity persists across diverse spatial scales and materials. The mechanistic basis for this universality was recently attributed to evolutionary selection for ease of piercing [H. Quan *et al*., *Proc. Natl. Acad. Sci. U.S.A.* **121**, e2316320121 (2024)]. However, the transient nature of their morphology, progressively modified by repeated use and inevitable wear, has received little scrutiny. In this work, we combine tabletop experiments with continuum analysis to demonstrate that the universal tip morphology can result from stochastic weathering processes. This finding is particularly significant in light of recent observations of the same tip geometry on dissolving or melting solids and geomorphic structures in addition to biological stingers. Our results suggest that the prevalence of this power-law profile may not be the result of evolutionary selection, but rather an inevitable consequence of exposure to random erosive processes.

## Significance

Pointed objects such as stingers, horns, and teeth have been observed to exhibit a paraboloid geometry at the tip. Interestingly, this tip geometry is not exclusive to biological structures; it is also found in abiotic forms as disparate as icicles and rock pinnacles. However, the conformity of tip shapes in biostingers has recently been selectively attributed to evolutionary convergence. In this work, we show that pointed tips of biological origin acquire the ubiquitous tip profile just as their abiotic counterparts are—by mechanical wear and usage. Our findings also explain the persistence of self-similar shapes observed in nonconical stingers, such as shark teeth and horns.

**Fig. 1.**
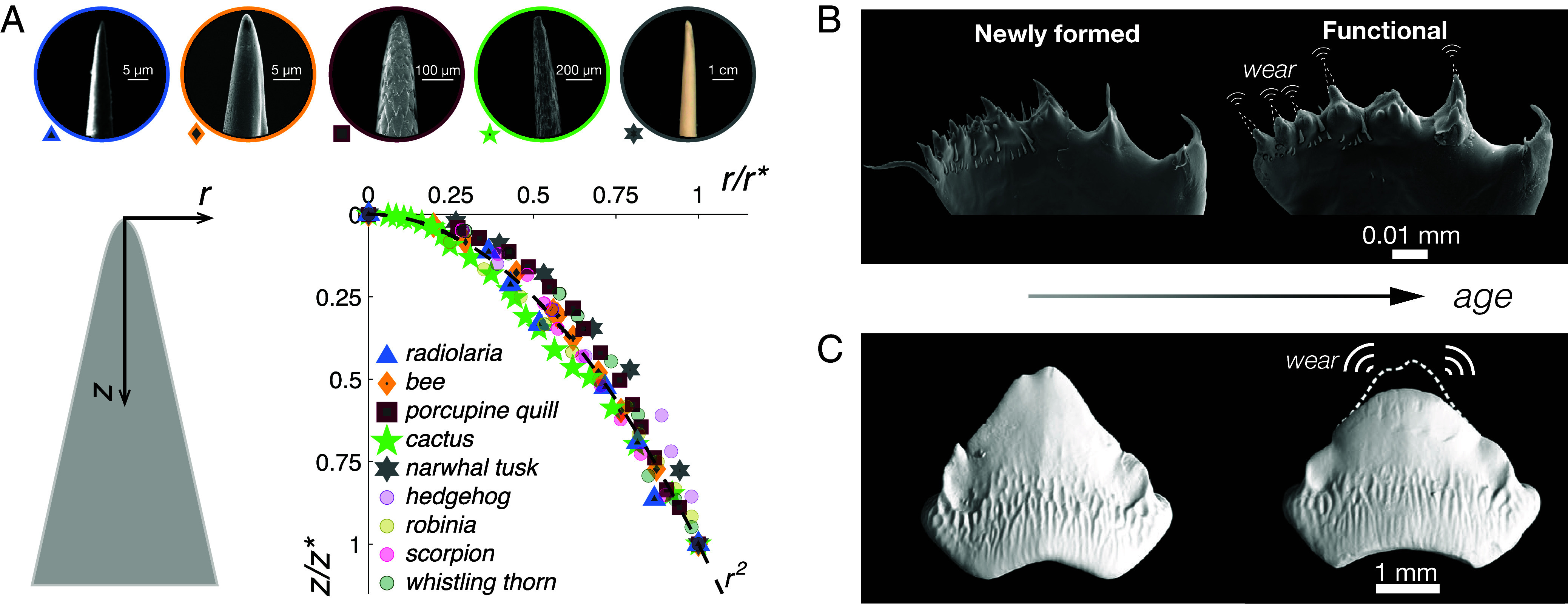
Examples and counterexamples of stingers and spikes exhibiting the ubiquitous tip profile $z∼r^{2}$ in biology. (*A*) Universal tip morphology of biostingers: Biostingers are observed to follow a conical form with a blunt apex. The radial (r) and axial (z) coordinates are centered at the apex in the illustration. Near the tip (as z→0), biostingers spanning a broad range of spatial scales and taxa exhibit adherence to a power-law profile described by $z∼r^{n}$, where n≈2. The plot presents cross-sectional tip profiles of axisymmetric biostingers, reproduced from data by Quan et al. (3). The coordinates r and z are each normalized by their respective maximal dimensional extents for each biostinger, denoted $r^{*}$ and $z^{*}$ (*SI Appendix*, Table S2). The prevalence of the parabolic tip profile, $z∼r^{2}$ was recently attributed to evolutionary optimization for efficient penetration into soft tissue (3). (*B*) Counterexamples in biology: Observations of such pointed structures over time, however, show that in their pristine or unused state, they have sharp tips. Scanning electron microscopy (SEM) images of copepod (*T. longicornis*) mandibles prior to feeding (*Left*) posses spinose tips. These initially conical tips are eventually worn by repeated use over time, and exhibits the universal profile when observed at a late lifecycle stage (*Right*), after several feeding cycles (8). (*C*) Alternative strategies to retain sharp features in Nature: Predatory animals such as sharks exhibit a preference for sharp teeth profiles and make use of multiple rows of “fresh” teeth to replace worn, blunted, or damaged teeth. The pristine teeth are protected from wear beneath an epithelial sheath. X-ray microtomography of such a tooth of an adult male Port Jackson shark from a row of pristine (*Left*) teeth gets worn over time and exhibits a persistent tooth shape (*Right*) (11). Eroded teeth are observed to preserve their general geometric form, even as their sharpness diminish, and are no longer functional. See also *SI Appendix*, Fig. S6, an image of the jaw of a nurse shark showing the typically hidden, inner layers of sharp teeth, and outer, exposed rows of teeth blunted by use (9). (Data and images in A: reproduced from ref. 3, CC-BY-NC-ND, PNAS 2024; Images in B: from ref. 8, CC-BY, PNAS 2024; Images in C: from ref. 11, CC-BY, Springer Nature 2020).

Pointed, spine-like structures or stingers are straightforward defense mechanisms ubiquitous in Nature, observed in organisms spanning a wide range of spatial scales and taxa (1, 2). High-resolution profiling shows that their conical apices consistently obey a power-law relationship, $z∼r^{n}$, where n≈2 (1.71<n<2.71), as reported by ref. 3 and directly reproduced in Fig. 1*A*. Such morphological relations have also been reported for a larger class of pointed biostructures such as teeth, mandibles, and horns (4). Recently, Quan et al. (3) attributed the morphological conformity of biostingers across organisms to evolutionary selection pressures favoring optimal penetration into soft substrates: Such a power-law profile minimizes tip buckling prior to penetrating into a soft substrate.

However, a broader inspection of pointed morphologies in Nature shows that the above universal tip profile is not exclusive to biostingers and occurs widely in the abiotic world. Fluid-mediated erosion of pinnacles (5), icicles (6), and dissolving cylinders (7) all drive axisymmetric bodies toward the same power-law profile observed in the previous examples from the biosphere, despite the disparate physical processes involved. In this light, it is important to isolate the role of such physical mechanisms morphing the true “design” of biological tip shapes, converged upon by allied evolutionary processes.

Differentiating the effects of such mechanical processes requires examination of biological tips from early stages in their life cycles, focusing on pristine structures that have not yet been deployed for their intended functions. However, the aforementioned studies on biological stingers have relied on samples collected at arbitrary points within an organism’s lifespan. A representative counterexample illustrating the transition from an initial conical, “spinose” geometry to the universal tip profile is provided by copepod mandibles, shown in both their pristine (Fig. 1*B*, *Left*) state before feeding and a subsequently worn (Fig. 1*B*, *Right*) state (8). Higher organisms such as sharks (e.g., *Heterodontus portusjacksoni*, shown in Fig. 1*C*, and *Ginglymostoma cirratum* in *SI Appendix*, Fig. S6) and dogfish (e.g., *Mustelus canis*) rely on the sequential replacement of exposed teeth, worn out or damaged by use, with new rows of sharp teeth (polyphyodont) preserved beneath protective epithelial layers (9, 10). These observations suggest that the impact of mechanical wear interactions on the shape of these biological tools are nonnegligible. As their morphologies change drastically over the typical lifespan of these organisms, the role of abrasion due to wear on biostingers must be accounted for, before conclusions relating to evolutionary “design” can be derived.

In the following sections, we use model experiments and a minimal theoretical model to demonstrate that stochastic weathering alone suffices to reproduce this universal morphology, $z∼r^{2}$, irrespective of material composition or structure, from biological stingers to geomorphological spikes.

## Experiments and Observations

**Fig. 2.**
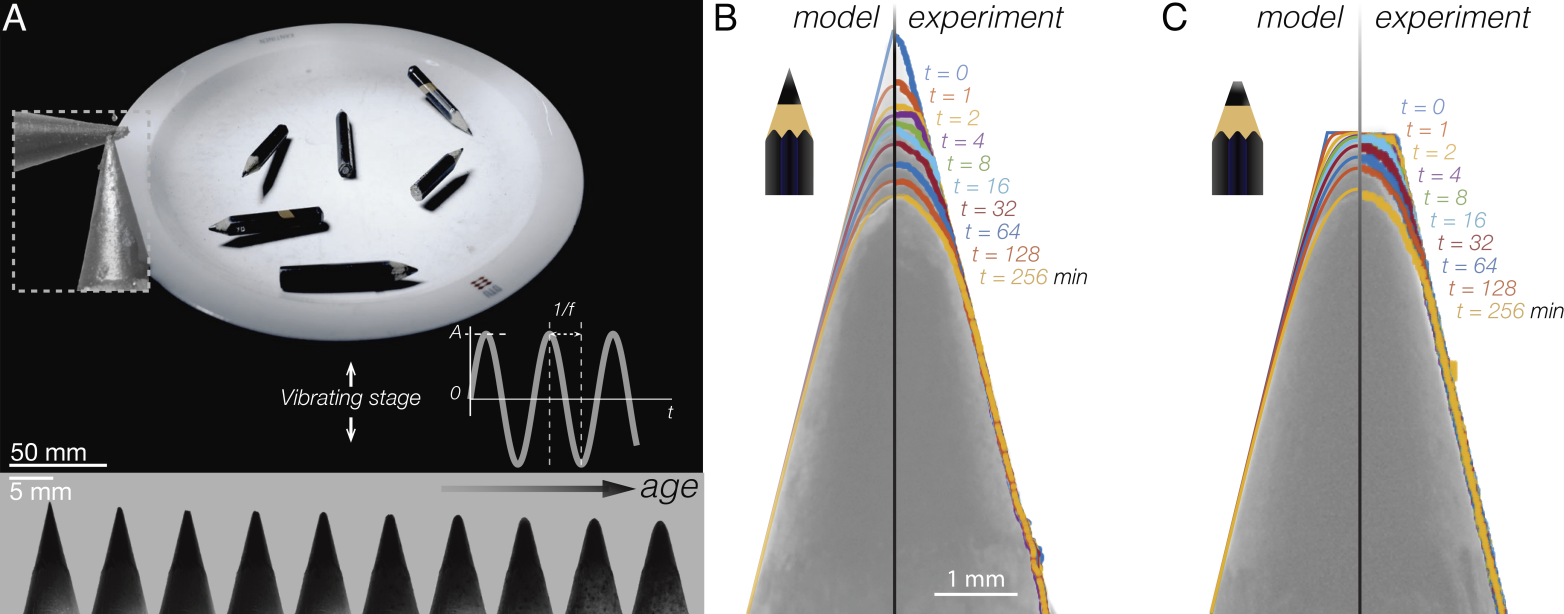
Experiments simulating stochastic wear interactions on pencil simulacra. (*A*) Experimental setup: Pencils sharpened to an initial conical profile are used as biomimetic stingers. They are placed on a porcelain plate atop a vibrating stage imparting kinematic forcing on the plate at a constant frequency f≈15 Hz and amplitude A≈2 mm. A stroboscopic photograph of the setup captures it in action. Vibration induced collisions mimic random wear on individual pencil tips. One such collision event is presented in the *Inset* (*Top Left*). See also Movie S1 and *SI Appendix*, Figs. S1–S3. Temporal evolution of the tip profile of each stinger is recorded optically at logarithmic time intervals, at timesteps t=0,1,2,4,8,16,32,64,128, and 256 min. A sequence of these images (*Bottom Inset*) offers a qualitative picture of how wear transforms tip geometries. (*B*) Geometric data of shape evolution: Pencil of grade 7B, with a sharp conical initial geometry at t=0 min. Coordinates of its boundary are extracted from optical data over time and are overlaid on the right half plane an image stack depicting the blunting process. A minimal dynamical picture of material removal by random wear is captured by Eq. **1**, numerical solutions to which are presented on the left half plane of the image stack, corresponding to experimental timesteps. After an initial phase of rapid fragmentation, the pencil tips conform to a parabolic profile. (*C*) Late time tip profile is independent of initial shape: Experiments are repeated on pencils of grade 7B with a different starting shape—that of a truncated cone. Geometric data from experiments (right half plane) and numerical solutions for the flat-tip initial shape (left half plane) are overlaid on image stack. At later time steps, the tip profile is similar to the late time shapes of initially sharp pencils.

To isolate the role of physical mechanisms in shaping the sharp-tip geometries, we choose sharpened pencil tips as model stingers, which are then exposed to stochastic abrasive events. This is achieved by placing sharpened pencils (diameter ∼7 mm, length ∼60 mm) upon a vibrating plate forced at a constant frequency (∼15 Hz) and amplitude ∼2 mm (Fig. 2*A*), inducing random collisions between pencils, including at multiple higher and lower frequencies, which lead to gradual material removal, thereby mimicking environmental wear processes encountered by natural stingers (see *Materials and Methods* for experimental details). Movie S1 illustrates material removal during pencil-pencil collisions (*SI Appendix*, Figs. S1–S3). The above parameters of vibrational forcing are chosen such that random material removal is constrained to the surface by “soft interactions” and that individual collisions do not cause bulk fracture events. Using pencil tips as simulacra provides several experimental advantages: availability of a material with uniform, isotropic material properties, ability to systematically vary material properties via alphanumeric grades (*SI Appendix*), flexibility in imposing initial shapes, and straightforward compliance with geometric constraints characteristic of natural stingers—namely, axisymmetry and overall right circular conicity.

The experiment tracks the evolution of pencil tip geometries using optical imaging over a timespan sufficient to observe convergence to stable profiles, recorded at logarithmic time intervals. Material removal rate depends on the mean frequency and energy of collisions, set by the vibrating plate’s frequency and amplitude. Three primary modes of kinematic exchanges occur: pencil-pencil collisions, pencil-plate impacts, and impact-induced internal shock waves. To determine the dominant mechanism for material removal, we conducted control experiments: one, with tips shielded by a protective cap to prevent direct contact; two, single-pencil tests to isolate the effects of pencil-plate impacts. These tests revealed that direct pencil-pencil collisions predominantly drive material removal within the overall timespan of each experiment (∼256 min). Videographic and direct visual inspection reveal that pencil bodies predominantly collide in roughly parallel orientations, whereas interactions involving pencil tips occur at various arbitrary Euler angles. An individual pencil experiences ∼5 such angled collisions per minute, randomly distributed in time, and as many as ∼15 in occasional bursts. No preferred collisional orientation could be observed; unsurprisingly, zero head-on collisions were spotted.

Each tip undergoes rapid fragmentation in the early stages, where arbitrary irregular intermediate tip shapes appear, followed by a slower blunting phase, where the tip shape evolves in a self-similar manner, as shown in Fig. 2*B*. To demonstrate that the convergent universal morphology is independent of esoteric early time shapes, experiments were conducted with flat, truncated tip as initial shape (Fig. 2*C*).

**Fig. 3.**
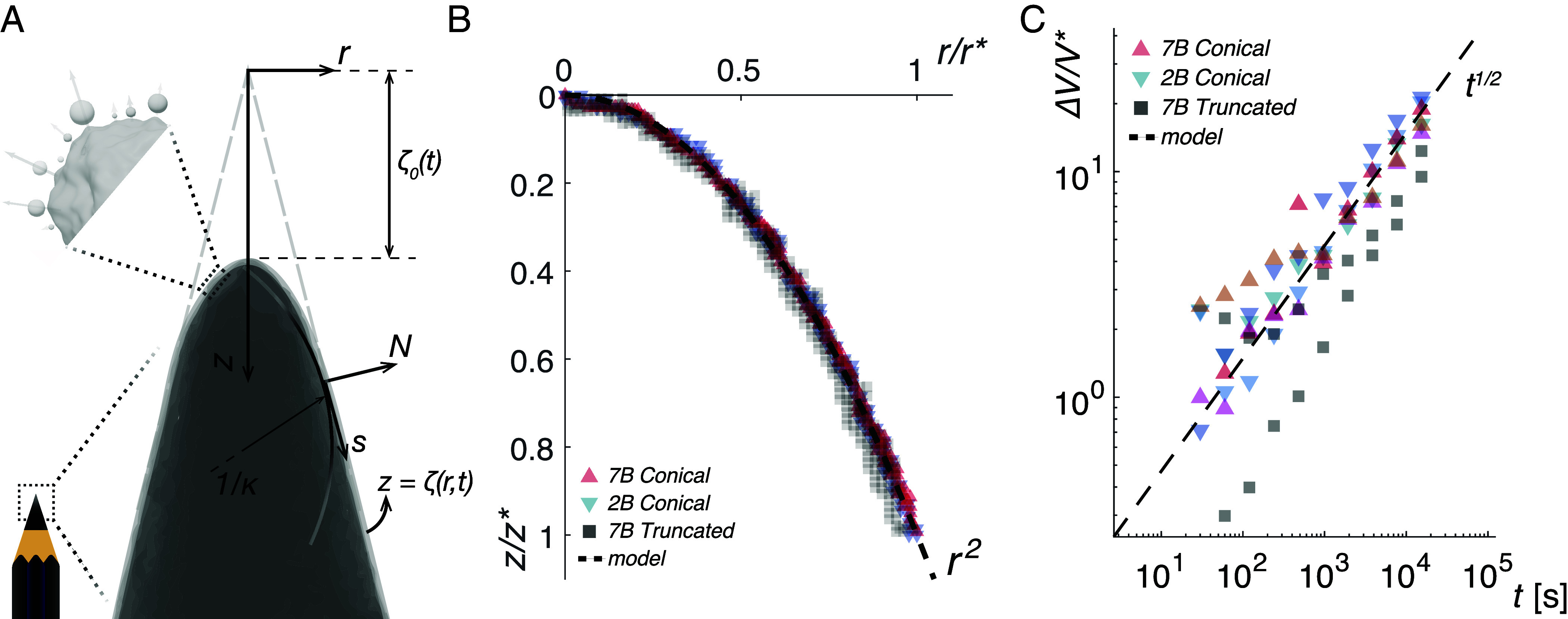
Material removal by mechanical wear shapes stinger tips to the ubiquitous profile $z∼r^{2}$ regardless of initial tip geometry and material composition. (*A*) Schematic illustration of random wear: An axisymmetric conical body with a blunted tip is depicted by its cross-section. Radial, r, and axial, z, coordinates are centered at the sharp tip of the extrapolated cone. The instantaneous boundary of the stinger is captured by the radius function, z=ζ(r,t), and the position of its apex at any instant is denoted $\zeta_{0}\left(t\right)=\zeta \left(0,t\right)$. At any point on ζ(r,t), the intrinsic arc length coordinate and local normal are denoted by s and N, respectively. Contact induced wear removes material in proportion to the local mean curvature of this interfacial surface, κ(r,t), and along the local normal. This principle is captured by Eq. **1**, which governs the temporal evolution of ζ(r,t). (*B*) Convergent tip shape is independent of initial shape and material composition: The tip profile of three pencil simulacra after being exposed to 256 min of wear interactions—$\zeta \left(r,t\right)-\zeta_{0}$ at t=256 min, are plotted. The three samples differed in initial geometry (conical or truncated) or pencil grade (2B or 7B). All samples converged to the same normalized profile, $z∼r^{2}$, in exact congruence with the biostinger profiles presented in Fig. 1*A*. This points to the possibility that a stinger observed to follow the ubiquitous parabolic profile at an arbitrary point in time, could have possessed a different initial or “pristine” morphology. (*C*) Cumulative volume loss over time: Volume lost to wear, ΔV(t)=V(0)−V(t) is plotted as a function of time for the three samples. The amount of material removed is dependent on material properties, namely the internal binding energy density of the tip material. Volume of material removed, ΔV, for each sample is normalized by, $V^{*}$, the volume lost by the softer pencil (7B) for the least amount of imposed vibrational energy times the ratio of the pencils’ binding energy density (*Materials and Methods*). Material removal follows a sublinear power-law in time, $\Delta V\left(t\right)∼t^{1/2}$, as predicted by the analysis of Eq. **1**.

The normalized tip profiles of 2B and 7B pencils with initially sharp (conical) and flat (truncated) geometries after 256 min of collision activity are presented in Fig. 3*B*, where coordinates are normalized by the respective coordinates of the points where the tip parabola tangentially meets the line representing the bounding cone. From these profile measurements, it is immediately evident that the conical tip is blunted by collisions to a shape not inconsistent with a power-law profile, $z∼r^{n}$, where the best fit (*Materials and Methods*) to data yields n=2±0.0093, in exact congruence with the biostinger morphologies presented earlier in Fig. 1*A*.

As noted earlier, this late-time profile is independent of both pencil grade (material properties), and initial shape. To verify the robustness of using pencil tips as simulacra, experiments were constructed with a pencil analogue, a cylinder with a conical apex, machined out of a natural bull horn (*Bos taurus*), colliding with slices of the same material with arbitrary shapes. To counterpoise the superior mechanical properties of these materials and resultant slow wear, the parameters of vibrational forcing were raised (see *Materials and Methods* for details). After ∼480 min of random collisional activities, the bull horn tip was observed to have converged to the expected parabolic profile (*SI Appendix*, Fig. S5).

Having experimentally demonstrated that stochastic weathering processes are sufficient to reproduce the omnipresent power-law profile of biostingers and numerous abiotic pinnacles, we shall now consider a simple dynamical picture of the above process of shape evolution driven by mechanical wear.

## Physical Mechanisms and Model

Pointed structures are employed for myriad functions in biology—from teeth or mandibles for mechanically breaking down food to stingers which are predominantly for defense or deterrence (1). Passive stingers typically remain attached to the organism, familiar examples being cacti needles, porcupine spines, and diatom spikes. In contrast, insect stingers and proboscises have an intended “active” end use. Regardless of their intended purpose and mode of deployment, all structures are exposed to their environments, and consequent mechanical wear over different spatial and temporal scales. In our experiments emulating such interactions on pencil simulacra, vibrational kinematics imposed by the plate induce pencil-pencil collisions distributed stochastically over time and acting at arbitrary positions on the material interface, and collectively exhibit all major mechanical wear modes—adhesive, abrasive, and fatigue (12). Material is worn off from the outermost surfaces, constrained by the overall geometry of the stinger, as illustrated in Fig. 3*A*. The geometry of these stingers which are axisymmetric around the z axis can be expressed by the radius vector r=(r(s,t),ζ(s,t)), z=ζ(r(s),t) being the radius function, where r and s respectively denote the radial and arc length coordinates, and t represents time. The evolution of shape of the stinger can therefore be envisioned as deformations of the geometric interface ζ(r(s),t), and can be formulated as a free boundary problem.

Over this geometric envelope, material is removed locally only along the outward normal and is not transported or relocated tangentially along the surface. Neither does material get deposited back onto the interface. Thus, the process is akin to related processes of chemical etching where weakly bound particles, inversely proportional to their coordination number, are removed at higher rates (13), regardless of the exact nature of the forcing that leads to wear, be it abrasion due to direct contact or weakening of internal binding by attrition. A coarse-grained view of this microscopic picture corresponds to the simple transport rule that the interface recedes in proportion to its local curvature (14). Adopting this model of stochastic material removal, the evolution of the outer surface represented by r(s,t) can be rewritten as $\partial_{t}r\left(s,t\right)=-D\kappa N$, where κ(s,t) and N(s,t) are respectively the local mean curvature and outward normal to the curve (Fig. 3*A*), and D is a constant of proportionality. It states that protrusions, or features with the highest local curvature, have the highest probability of contacting one another in our collision experiments, which entails our intuitive expectation that sharp features are the quickest to wear out, instantiated by the everyday experience that the tip of newly sharpened pencil is easier to break off. This view corresponds directly with the physical process emulated in our experiments. Since material removal rate varies linearly with contact stresses (12), the probability of local material removal is determined by the relative orientation of colliding pencils. Given the stochastic nature of vibrational forcing, the relative orientational phase space can be considered ergodic, whereby collision probability at any given point on the object is simply proportional to its relative proximity to the colliding neighbor or surface.

Recognizing that our model is the geometric diffusion equation (15), the above equation is equivalent to

[1] $$\begin{array}{c} \partial_{t}\zeta =D\partial_{\mathrm{ss}}\zeta , \end{array}$$

where D plays the role of an apparent diffusivity, dependent on the material properties and collision frequency, often referred to as a form of surface tension, or, energy density on the interface (16). This approach is also similar to classical treatments of geomorphic evolution over large spatial and temporal scales driven by stochastic weathering (17). Furthermore, it offers yet another interpretation of random wear. The growth of solid interfaces due to random deposition of materials is similarly modeled using the Edwards-Wilkinson framework (18), which captures the notion that locations of concavities with the highest curvature on a surface have the highest probability to be filled. This is an antiparallel to the process of erosive material removal described above. Indeterministic, random material removal can therefore be envisioned as the inverse process of random material deposition over a surface, captured essentially by the same mathematical model. We, therefore, adopt this framework, albeit with the following simplifications. One, while it is customary to capture local variations and indentations using higher-order terms, we will disregard higher derivatives and explicit descriptions of noise in the subsequent analysis, since we are interested only in the mean geometry of the interface. Two, unlike the classical random deposition model which is often applied to horizontal initial geometries, we consider surfaces of revolution, whereby the local tangential coordinate is the arc length, s, and the harmonic operator is the intrinsic Laplacian.

This formulation of random wear as a free boundary problem, where the generator curve or radius function ζ(r(s),t) “shrinks” according to Eq. **1**, is referred to as geometric curve shortening or mean curvature flow (15, 19, 20), following standard terminology in differential geometry. Motivated by our experimental observations that no prominent concavities emerge during the evolution of tip morphology, we regard the overall geometry to remain convex. This assumption is consistent with well-known results in differential geometry (15, 21), which show that the development of significant concave features leads to pinch-off events, after which the remnant geometries proceed to evolve by mean curvature flow, permitting the use of Eq. **1**, without modifications, for the analysis below, and numerical solutions (presented in Fig. 2 *B* and *C*).

Prior to solving Eq. **1** for specific geometries of interest, it is prudent to validate the model against a measurable quantity—the cumulative volume removed from each stinger simulacrum, ΔV(t)=V(0)−V(t). The Green’s function corresponding to Eq. **1** is given by $G\left(s-s_{0},t\right)={\left(4\pi Dt\right)}^{-1/2}\exp\left(-{\left(s-s_{0}\right)}^{2}/4Dt\right)$, which scales as $t^{-1/2}$ following a short initial phase dominated by an exponential fall. Geometry of the stinger at a later time, t, given by $\zeta \left(s,t\right)=\int G\left(s-s_{0},t\right)\zeta_{0}\left(s_{0}\right)\;ds_{0}$, therefore follows suit, advancing for long times as $\zeta \left(s,t\right)∼t^{-1/2}$. The corresponding volume of revolution is $V\left(t\right)=\pi \int r^{2}\left(s,t\right)\partial_{s}\zeta \left(r,t\right)\;ds$. Since $\left(s-s_{0}\right)∼t^{1/2}$, it follows that $r^{2}\left(s,t\right)∼t$ and $\partial_{s}\zeta \left(s,t\right)∼1/t$, leading to $V\left(t\right)∼t^{1/2}$. Therefore, the late time behavior of material removal can be expected to follow $\Delta V=V\left(0\right)-V\left(t\right)∼t^{1/2}$. We plot experimental ΔV(t) for 7B and 2B pencils with conical and truncated conical initial shapes in Fig. 3*C* and see that it adheres to the predicted scaling after an initial phase of rapid fragmentation. Having validated the minimal theoretical model against experimental outcomes, we now solve Eq. **1** numerically for initial geometries considered in this work—a cone and a truncated cone. The temporal evolution of these shapes are consistent with experimental data, as shown in Fig. 2 *B* and *C*, where numerical solutions corresponding to experimental time steps are presented.

Thus, the minimal dynamical model considered above suffices to explain both of the main experimental measurements–1) coordinates or geometry of stingers at different time steps (Fig. 2 *B* and *C*), and 2) cumulative volume loss over the span of the experiment (Fig. 3*C*). Emboldened by the agreement between numerical solutions of Eq. **1** and experimental data, we can now investigate whether the model also predicts the universal power-law profile, $z∼r^{2}$, close to the tip. To do so, we seek admissible solutions of the form $\zeta \left(r,t\right)∼\zeta_{0}\left(t\right)+\frac{1}{2}\kappa_{0}\left(t\right)r^{n}$ at the limit, r→0. Here, $\zeta_{0}\left(t\right)=\zeta \left(0,t\right)$ and $\kappa_{0}\left(t\right)=\kappa \left(0,t\right)$ are respectively the instantaneous position and curvature of the stinger tip at r=0. The exponent, n, imposes a power-law profile which merges tangentially with the outer cone. We begin by rewriting Eq. **1** in (r,z) coordinates

[2] $$\begin{array}{c} \partial_{t}\zeta =D\left(\frac{\partial_{\mathrm{rr}}\zeta }{1+{\left(\partial_{r}\zeta \right)}^{2}}+\frac{1}{r}\;\partial_{r}\zeta \right). \end{array}$$

Substituting the implied power-law tip and simplifying Eq. **2** for small r, we get $\partial_{t}\zeta =\partial_{t}\zeta_{0}+\frac{1}{2}\partial_{t}\kappa_{0}\left(t\right)r^{n}\approx \frac{1}{2}D\kappa_{0}\left(t\right)n^{2}r^{n-2}$. Matching powers of r, we see that a nontrivial solution exists only for n=2, for which the rate of tip descent, $\partial_{t}\zeta$, is finite (*Materials and Methods*). Thus a stinger has the geometric form of a base cone with its tip rounded to follow $\zeta \left(r,t\right)-\zeta_{0}\left(t\right)∼r^{2}$, regardless of its exact initial shape and material properties. We plot the late-time profiles, $\zeta \left(r,t\right)-\zeta_{0}\left(t\right)$, of 7B and 2B pencils starting with an initial conical profile and 7B pencils starting as a truncated cone after 256 min in Fig. 3*B*, which is in agreement with the above solution. The sublinear power-law of volume loss reiterates that this stable shape persists, even as the tip position, $\zeta_{0}\left(t\right)$, and its sharpness, $\kappa_{0}\left(t\right)$, evolves, until all material is eventually removed as t→∞. The slow progression of wear blunts the stinger tip, observed as an increase in the radius of tip curvature (*SI Appendix*, Fig. S7) or equivalently as the widening of the parabolic tip. Furthermore, it suggests that intermediate tip profiles for which n≉2 will erode rapidly, raising the likelihood that a biostinger observed at an arbitrary intermediate point of their lifespan will exhibit the above parabolic tip morphology even as it gets duller, i.e., the prefactor or relative tip sharpness, $\kappa_{0}$, diminishes.

The expression for the progression of $\kappa_{0}\left(t\right)$ can be found by substituting the above tip profile (Eq. **7**) into the governing Eq. **2** and is given by $\kappa_{0}\left(t\right)=\kappa_{0}^{*}/\sqrt{1+4D{\kappa_{0}^{*}}^{2}\left(t-t^{*}\right)}$, where, $\kappa_{0}^{*}=\kappa_{0}\left(t^{*}\right)$ is the tip curvature at a previous time of observation, $t^{*}$ (see Eqs. **8** and **9** in *Materials and Methods*), in agreement with experimental data presented in *SI Appendix*, Fig. S7. Therefore, at long times $\kappa_{0}∼t^{-1/2}$, from which it indeed follows that the rate of volume loss, $\Delta V/\Delta t\propto \kappa_{0}$, consistent with the central mechanistic hypothesis that geometric erosion rate is proportional to local curvature. In particular, the transient length scale associated with wear-driven morphing, the radius of tip curvature, $1/\kappa_{0}=\sqrt{4Dt}$ over long times.

Conversely, the characteristic time for tip wear scales as $\tau_{w}∼1/D\kappa_{0}^{2}$. The time scale depends on the apparent diffusivity, D, whose value reflects both the mean energy of interactions and the mechanical properties of the stinger and the materials with which it interacts. For pencils of different grades (2B and 7B) studied under identical experimental protocols, the corresponding values of D are estimated using Eq. **9**, and are stated in Table 1. Interestingly, we find that $D_{2B}\approx \frac{2}{7}D_{7B}$, which coincides with an inference on their relative binding energy densities from independent pencil truncation experiments that $e_{2B}\approx \frac{2}{7}e_{7B}$ (*Materials and Methods*). This correspondence provides further support for the applicability and validity of the model in capturing the effect of random wear on geometric flow. Accordingly, mapping tip curvature over time offers a convenient, minimally invasive means to characterize the progression of tip wear in biostingers in situ, and to estimate the apparent diffusivity, D, for natural processes (*SI Appendix*, Fig. S7). The latter serves as a cumulative marker of stochastic mechanical interactions, though often neglected, as we have shown, play a nonnegligible role in shaping the natural world.

**Table 1.** Geometric and material properties of Grade B (Soft Black) pencils

Grade	Core radius [mm]	Truncation height [mm]	Removed volume *δV* [mm^3^]	Graphite %	Diffusivity *D* [mm^2^/s]
2B	2.0	1.0063	0.0646	74	0.003
7B	3.2	1.4842	0.2326	87	0.011

## Discussions and Conclusions

Close to their tips, stingers, spikes, mandibles, which are roughly of conical form, are observed to follow a power-law profile, $z∼r^{n}$, where n≈2. While the ubiquity of this shape in biology was attributed to evolutionary optimization for efficiency of penetrating or piercing into soft tissue, we present counterexamples in biology which indicate that in their pristine state, prior to ever being exposed to mechanical interactions, they retain sharp geometries which are intuitively associated with polearms. Moreover, several organisms employ alternative mechanisms, mainly—the replacement of worn stingers. Therefore, we explore how this mechanical component, namely, random wear, affects stinger morphology using pencil tips as simulacra. We show that, all stinger samples, regardless of their initial shape or material composition, are eventually shaped to follow $z∼r^{2}$, a parabola which is tangentially pinned to the bounding cone. Material removal by such stochastic forcing at their instantaneous boundaries was captured using a minimal dynamical model posed as a free boundary problem, which retrieves the observed tip shape as a stable solution.

As we have seen, there are few instances where a pointed tip—biostingers, mandibles, horns, chosen arbitrarily from Nature—will exhibit a shape that deviates from the observed universal power-law. In fact, numerous erosive mechanisms result in the same tip profile even when constrained by different boundary conditions. Together with the observation that structural overdesign is rare in Nature, this suggests that natural stingers are generally susceptible to, rather than protected from, habitual wear. An illustrative example is provided by the pristine siliceous mandibles of copepods shown in Fig. 1*B*, which are predominantly used to feed on other siliceous forms, such as diatoms. More broadly, stingers composed of softer tissues tend to interact primarily with comparably soft materials, whereas at the opposite end of the spectrum, large animals such as elephants possess ivory tusks that predominantly interact with hard substrates, including elephant hide and tree bark.

A corresponding description is afforded in terms of the characteristic time scale for wear, $\tau_{w}∼1/D\kappa_{0}^{2}$. Naturally, this time scale is small for small organisms owing to high values of $\kappa_{0}$. However, assuming that $\tau_{w}$ is much smaller compared the lifespan of a given organism, the magnitude D can be expected to play a compensatory role. For instance, the lifespan of pencil stingers in our experiments is several tens of hours, whereas $\tau_{w}$ is of the order of minutes. In this light, it may be postulated that combinations of material composition, size, and organismal activity observed in Nature are those which collectively enhance D, such that for small organisms with stingers made of soft materials this time scale may span hours to days, while for large organisms $\tau_{w}$ may extend to several months or years. In an evolutionary sense, these stochastic, but recurrent interactions likely contribute to setting the optimal material properties of stingers suited to each organism. For herding animals, the largest contributor to such mechanical interactions are between horns/ tusks and the hide or skin of fellow organisms, the material properties of which have been qualitatively correlated to that of their stingers (22). A possible avenue to evaluate such a convergence in material properties, and deviations therefrom, is to study the effects of mechanical interactions on organisms existing outside their natural habitats—for instance, in urban settings, in comparison to their wild conspecifics (23).

Our experiments mimic repeated “soft” interactions between materials of similar properties in a manner conducive to repeatable, uninterrupted dynamic forcing over long times. Meanwhile, stingers are typically studied using classical penetration experiments involving quasi-static directional loading into a soft substrate (3, 24). Drawing upon the finite element simulations of the stress field surrounding a piercing stinger by Zhang et al., (24) (see Fig. 4F therein), we note that the stresses acting upon the surface of the stinger is approximately uniform, independent of the direction of loading, and scale with the shear modulus of the substrate material. This is characteristic of soft materials, which can accommodate large local strains. Moreover, biostingers are employed in a dynamic manner in Nature (21), further enhancing dynamic abrasive interactions. Since our model is indifferent to the exact details of the origin of external stresses acting on the stinger surface, and captures the mechanistic principle that weakly bound particles are the quickest to wear out, it is expected to remain applicable for these cases as well. However, many biological structures possess complex hierarchical or laminated architectures, for which surface properties may evolve anisotropically during wear, potentially necessitating model extensions. Nonetheless, as fragmentation and abrasive material removal are history-independent processes even for laminate structures (25), such extensions may be accommodated through a spatially dependent effective diffusivity, D(r), which consequently may lead to tip profiles that deviate from the currently observed parabolic profile, i.e., n≉2.

While the prevalent “smooth” shape can rightfully appear counterintuitive for the purpose of imposing damage on some soft material substrate—chewing to piercing—unlike sharp geometries, they are less susceptible to damage. A reduced likelihood of future damage may be more preferable for organisms than possessing the most efficient stinger for defense. Such an interpretation was offered by Darwin (26) upon observing how large felines scrubbed their claws to a smooth shape. Similarly, biostingers composed of live tissue, such as in plants, may be shaped by sequential exfoliation of their outermost layers (27). Even shark teeth, which are blunted and replaced in a span of weeks, were observed to retain their overall geometric form despite losing their sharpness (tip curvature) over time (11). This was, however, attributed to the microstructure and layered architecture of the shark tooth, even though its material properties were seen to have minimal role in its mechanical performance. Our work suggests, instead, that the self-preserving (self-similar) shape emerges naturally when the roughly hemiellipsoidal tooth is exposed to wear, regardless of its material properties, and exact initial tip shape. Therefore, the framework of wear-driven shaping, illustrated here for axisymmetric stingers, may apply more broadly beyond stingers and teeth to other biological structures, such as beaks and horns, for which wear is a natural consequence of their functional roles. However, the paucity of available data currently limits such an analysis. Such is the case also for organisms with concealed stingers, such as certain bees and wasps, as well as for everting organisms such as bloodworms, whose pointed appendages primarily interact with surrounding soft substrates, and the extent to which exfoliatory events ensue remains unknown.

Nontrivial biostinger shapes that deviate significantly from the “universal” profile can also be observed, but they usually correspond to damage or injury, and drastic collision events, where the energy of impact far exceeds the internal binding energy density of the stinger material. However, these high energy “hard interactions” produce rare but catastrophic events, leading to severe damage in both pencil specimens (*SI Appendix*, Fig. S3) and natural stingers (28). In contrast, wear interactions are those which result from mechanical events where the energy of impact is roughly of the order of the binding energy of the stinger material. These low-energy collisions constitute a subset of all mechanical events which may be referred to as “soft interactions,” encompassing those examined in our experiments with pencil simulacra. They result in exfoliatory material removal confined to the surface without inducing bulk responses such as plastic deformation or object-scale fracture. While worn shapes reminiscent of plastic deformation and bulk cleavage have been observed in a variety of museum samples, including the canine teeth of Tasmanian devils (29) and myriad marine organisms (30), data on the temporal progression of these morphologies remain scarce. Whether such shapes result from predominantly “soft” or “hard” interactions, or through the effect of other simultaneous chemical processes, can be ascertained only through dynamic testing and systematic tracking of shape evolution and is therefore left to future studies.

Though the overall form and initial, pristine geometry of pointed appendages in Nature result from diverse developmental pathways (28, 31), our results suggest that wear-driven mechanical shaping offers a rationale for the pervasiveness of the parabolic stinger tip profile observed at arbitrary later stages, regardless of initial shape, size, or material properties. That the resulting tip profile may be indifferent to any optimal functional role also appears unexceptional in a broader context, as several other geometric attributes of sharp appendages in organisms also do not seem to be of relevance in the light of efficient piercing. For instance, the degree of curvature of curved biological puncture tools such as fangs, claws, and stingers along their central axis has been shown to have minimal impact on their ability to penetrate soft tissue and to induce damage (24). Furthermore, the reasoning proposed by Quan et al. (3) for the existence of power-law profiles of the form $z∼r^{n}$ applies equally well for all exponents in the range 2≲n≲6 (see figure 3 of ref. 3). However, as they noted, the vast majority of biostingers exhibit n≈2. The often overlooked route of erosive shaping we have reproduced here using stinger simulacra presents an equally plausible explanation for the existence of the observed universal morphology, which suggests that stable shapes that are less prone to future rapid damage follow $z∼r^{2}$. That the universal profile also falls within the family of curves suitable for efficient piercing is perhaps an example of natural serendipity. However, as noted recently by Gayford et al. (32), even allometric relations in the natural world that hold across scales do not necessarily follow from underlying biological processes. In this vein, the existence of a universal shape that can be explained as an inevitable mechanical consequence of usage, but has the guises of an optimized shape, offers a cautionary tale in biomimetics and should inspire us to consider the effects that temporal, mechanical processes have on Nature’s “designs.”

## Materials and Methods

### Experimental Details.

The experiments simulate wear interactions on pencil tips (biomimetic stingers) by subjecting them to stochastic, low-energy collisions on a vibrating plate. A porcelain plate is placed upon a 120 W vibrator (JG-203, YILIKISS, Amazon Europe Core, Neudorf-Weimershof, Luxembourg). The inner diameter of the plate is 18 cm. Graphite pencils (Derwent, Cumberland Pencil Company, Workington, United Kingdom) of alphanumeric grades 7B and 2B were used. Pencils were sawed to smaller lengths of ∼6 cm each, roughly 1/3 of the plate’s inner diameter. The pencils were sharpened in multiple steps using an adjustable multiangle sharpener (HiLine T’GAAL, Kutsuwa Corporation, Higashi-Osaka City, Japan), set to angle position–3 corresponding to a cone angle of 27^°^.

The vibrating stage actuates the plate at roughly 15 Hz and an amplitude of 2 mm. Pencils which are free to move freely in all directions and orientations above the plate encounter collision activity at multiple higher and lower frequencies. The tip profiles were captured optically on a backlit background at logarithmic time intervals, t = 0, 1, 2, 4, 8, 16, 32, 64, 128, and 256 min. A 3 pixel wide band along the boundary was extracted. Profile data at different time steps were aligned using their linear conical base reference for each pencil. Exponents, n, were found by fitting each aligned profile data for late times to Eq. **3** by the native nonlinear least squares curve fitter in MATLAB. Following this fitting procedure on biostinger profile data from ref. 3—reproduced in Fig. 1*A*, gives n=2.082 and bounds [2.0005,2.1635] as opposed the range reported therein, which followed a log transformed linear fit, despite the relatively low number of data points (∼10), and therefore, in closer agreement with the parabolic tip profiles observed in icicles (33), pinnacles (34), and popsicles (7), each shaped respectively by the different mechanical erosive processes of melting, shear flow, and dissolution. Further, Fourier Cosine fit of order 20 were performed, capturing small local features on the profile, such as indentations, and then numerically integrated to compute the corresponding volume of rotation, V(t).

Experiments were repeated for a different initial geometry—truncated cone. These flat pencil tips were produced reliably using a craft cutting machine to trace out an Archimedean-type spiral of the form $r=r_{0}\theta^{1/4}$ for θ∈[0,70π] at a slow, constant speed. The total path length traversed by the pencil tip is therefore $s\left(70\pi \right)=\int_{0}^{70\pi }\sqrt{\frac{1}{16\theta^{3/2}}+\sqrt{\theta }}\;d\theta$, which corresponds dimensionally to 14.969 m. We used a Silhouette^®^ Cameo™ 5 craft cutter with a pen attachment and a 3D printed crown to add a mass of 133 g to the top of each pencil. The spiral was traced out on A4 250 GSM card stock paper at the lowest speed setting emulating a quasi-steady grinding process. We used pencils of grades 2B and 7B, the geometric and compositional details of which are tabulated in Table 1. The softer pencil, 7B, sheds more material than 2B. As the work done on each pencil in tracing out the spiral is the same, the volume of material removed, $\delta V_{i}$, is indicative of their respective internal binding energies, $e_{i}$, where i indicates numeric grade for soft (B) pencils. For a constant average energy, ⟨E⟩, imparted to the material as work done for material removal, $\left\langle E\right\rangle =e_{i}\delta V_{i}=\text{const}$. Notably, the ratio of volume removed – 2B-to-7B is $\delta V_{2}/\delta V_{7}=0.0646/0.2326\approx 2/7$, the ratio of their respective numeric grades. This information is used to normalize the volume of material removed, ΔV, in Fig. 3, defining $V_{i}^{*}=\left\langle E\right\rangle /e_{i}$. Since binding energy densities, $e_{i}$, are not directly measurable, we choose $V_{7B}^{*}=\Delta V_{7B}\left(t=60\;s\right)$ and $V_{2B}^{*}=V_{7B}^{*}\left(e_{7B}/e_{2B}\right)=\frac{7}{2}V_{7B}^{*}$.

Experiments were conducted on a pencil-analogue shaped from a typical biostinger, the bull horn (*B. taurus*). Disks of natural bull horn material of thickness 10 mm (Læderiet ApS, Denmark) were machined to the form of a pencil—a cylinder of diameter 10 mm with a conical apex of internal angle 50 deg and initial height ∼10 mm. The “bull horn pencil” was placed on the vibrating stage along with other wood encased graphite pencils and randomly shaped pieces of the same horn material to collide with. As expected, wear progresses much slower on the bull horn than with pencils. With an increased amplitude of ∼10 mm, and forcing frequency of ∼50 Hz, the bull horn took roughly four times the time taken for pencils to begin exhibiting the self-similar parabolic tip shape (∼6 h). We continued the experiments up to 480 min. Initial and final shapes are presented in *SI Appendix*, Fig. S5 *A* and *B*. Unsurprisingly, to the tip converged to the expected profile, that of a parabola pinned tangentially to the bounding cone (*SI Appendix*, Fig. S5*C*).

### Predicting the Power-Law Tip Profile from Eq. **2**.

To show that Eq. **2** ‘selects’ the exponent n=2 among all local power-law tip profiles, we consider the ansatz

[3] $$\begin{array}{c} \zeta \left(r,t\right)\;=\;\zeta_{0}\left(t\right)\;+\;C\left(t\right)\;r^{n}\;+\;O\left(r^{n}\right)\;\text{as}\;r\to 0, \end{array}$$

for some n>0 and coefficient C(t). Then

[4a] $$\begin{array}{cc} \partial_{r}\zeta & =Cn\;r^{n-1}+O\left(r^{n-1}\right), \end{array}$$

[4b] $$\begin{array}{cc} \partial_{\mathrm{rr}}\zeta & =Cn\left(n-1\right)\;r^{n-2}+O\left(r^{n-2}\right), \end{array}$$

[4c] $$\begin{array}{cc} {\left(\partial_{r}\zeta \right)}^{2} & =C^{2}n^{2}\;r^{2n-2}+O\left(r^{2n-2}\right). \end{array}$$

To ensure smooth axisymmetry at the tip, $\partial_{r}\zeta \left(0,t\right)=0$. By Eq. **4a**, this requires $\lim_{r\to 0}Cn\;r^{n-1}=0$; therefore, n>1, unless C≡0. Moreover, the term $\left(1/r\right)\;\partial_{r}\zeta$ in Eq. **2** behaves like $Cn\;r^{n-2}$; to be finite at r=0, we must have n≥2 for nontrivial C. Inserting Eqs. **4a**–**4c** into the right-hand side of Eq. **2**

[5] $$\begin{array}{c} \text{RHS}=D\left(\frac{Cn\left(n-1\right)r^{n-2}}{1+C^{2}n^{2}r^{2n-2}}+Cn\;r^{n-2}\right)+O\left(r^{n-2}\right)\\ \overset{r\to 0}{=}D\;C\;n^{2}\;r^{n-2}+O\left(r^{3n-4}\right).\;\;\;\;\; \end{array}$$

Similarly, the left-hand side is

[6] $$\begin{array}{c} \text{LHS}\;=\;\partial_{t}\zeta \;=\;\partial_{t}\zeta_{0}\left(t\right)+\partial_{t}C\left(t\right)\;r^{n}+O\left(r^{n}\right). \end{array}$$

Comparing Eqs. **5** and **6** as r→0, n=2 is seen to be the unique admissible exponent, where C is nontrivial, giving RHS $∼4DCr^{0}$, which is constant in r, and LHS $∼\partial_{t}\zeta_{0}+\partial_{t}Cr^{2}$. If n<2, the $r^{n-2}$ term diverges unless C=0; if n>2, the LHS term $\partial_{t}C\;r^{n}$ decays faster than the RHS term $DCn^{2}r^{n-2}$, so equality for all small r requires the coefficient of $r^{n-2}$ to vanish, i.e., C=0.

For n=2, $\zeta \left(r,t\right)=\zeta_{0}\left(t\right)+C\left(t\right)\;r^{2}+O\left(r^{2}\right)$, whereby $\partial_{r}\zeta =2Cr,\partial_{\mathrm{rr}}\zeta =2C$. Since the curvature of the axisymmetric generator curve is

$$\begin{array}{c} \kappa \left(r,t\right)=\frac{\partial_{\mathrm{rr}}\zeta \left(r,t\right)}{(1+{\left(\partial_{r}\zeta \left(r,t\right)\right)}^{2}{)}^{3/2}}=\frac{2C}{(1+4C^{2}r^{2}{)}^{3/2}}\;\overset{r\to 0}{\to }\;2C, \end{array}$$

C(t) is related to the tip curvature as $C\left(t\right)=\frac{\kappa_{0}\left(t\right)}{2}$. Thus, the admissible power-law profile for tip geometry, as shown in Fig. 3*B* is

[7] $$\begin{array}{c} \zeta \left(r,t\right)=\zeta_{0}\left(t\right)+\frac{\kappa_{0}\left(t\right)}{2}r^{2}. \end{array}$$

Substituting Eq. **7** into Eq. **2** and matching coefficients yields an expression for the evolution of tip curvature $\kappa_{0}\left(t\right)$

[8] $$\begin{array}{c} \partial_{t}\kappa_{0}\left(t\right)=-2D\;\kappa_{0}{\left(t\right)}^{3} \end{array}$$

the solution to which is

[9] $$\begin{array}{c} \kappa_{0}\left(t\right)=\frac{\kappa_{0}^{*}}{\sqrt{1+4D{\kappa_{0}^{*}}^{2}\left(t-t^{*}\right)}},\;\kappa_{0}^{*}=\kappa_{0}\left(t=t^{*}\right). \end{array}$$

## Supplementary Material

Appendix 01 (PDF)

Movie S1. Video sequence illustrating material removal during pencil-pencil collisions.

## Data Availability

Data in Figs. 1–3 have been deposited in GitHub (https://github.com/Jensen-Lab/sebastian2026.git) (35).
