# Supplementary material for: The geometry of Nature’s stingers is universal due to stochastic mechanical wear
Source: Proc Natl Acad Sci U S A. 2026 Mar 6;123(10):e2526098123. doi: 10.1073/pnas.2526098123 (PMC12974445; doi:10.1073/pnas.2526098123)
Supplement: Supplementary file 1 — Appendix 01 (PDF) [file pnas.2526098123.sapp.pdf]

# Supporting Information for

## The geometry of Nature's stingers is universal due to stochastic mechanical wear

**John Sebastian and Kaare H. Jensen**

**E-mail: khjensen@fysik.dtu.dk**

### This PDF file includes:

Supporting text
Figs. S1 to S7
Tables S1 to S2
Legend for Movie S1
SI References

### Other supporting materials for this manuscript include the following:

Movie S1

## Supporting Information Text

### 1. Birth, life, and death of a pencil tip

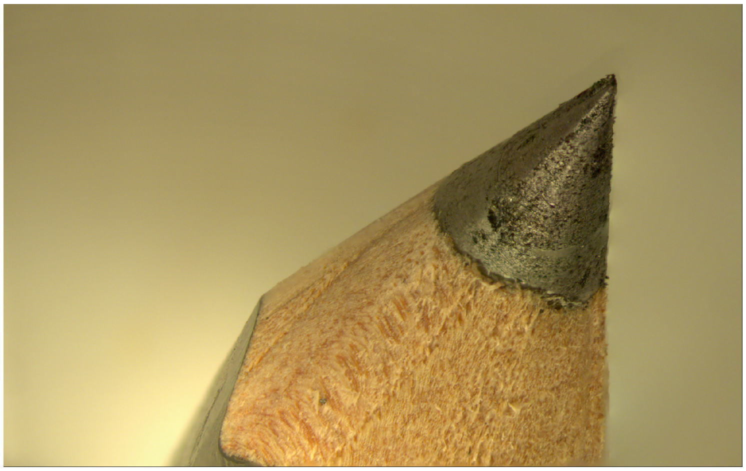

**Fig. S1. A freshly sharpened pencil tip**

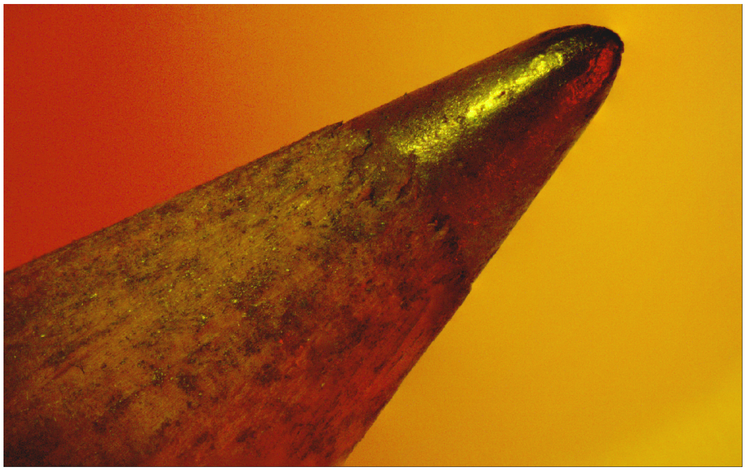

**Fig. S2. A pencil tip sculpted by random collisions**

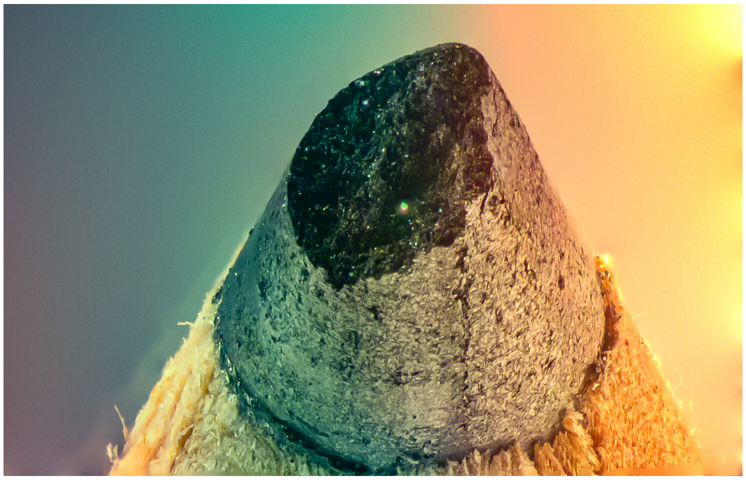

**Fig. S3. The tip of a pencil that was dropped on the floor**

## 2. The Pencil tip: an archetypal stinger

The use of pencil tips as stinger simulacra in this work is facilitated by their standardised properties imparted by their manufacturing process. The writing core or 'lead' of graphite pencils is manufactured via a high-temperature sintering process applied to a granular mix of graphite and clay (roughly 50%-50%) and a small amount of wax as a lubricant. The mechanical properties of the resulting core, and thus the grading of the pencil, are governed by the relative concentrations of graphite and clay in the mixture. Compositional details for different pencil grades obtained from Ref. (1) is tabulated in Table S1.

The graphite-clay 'dough' is shaped to the slender cylindrical form of the core by extrusion, after which it is baked in a sintering process at around $800 \deg C$ (2, 3). The radius of the core varies between 2 - 4 mm depending on its alphanumeric grade - softer-grade pencil leads are thicker so that the ergonomics of pencil use remain similar across grades (see Table 1 in Materials and Methods). The millimetric radii of pencil cores ensure that the thermal binding process is uniform across its cross section, yielding macroscopically homogeneous mechanical properties. The pencil cores are then left to cool naturally to room temperature.

Subsequently, the cores are placed into grooves on wooden panels with a pre-applied thin film of glue and allowed to bond. The grooves are pre-formed so that the wooden encasement does not impose compressive loads on the core or retain residual stresses. Following this step, individual pencils are cut from the panel and machined into their final hexagonal, circular, or other outer geometries.

**Table S1. Compositional details of pencil cores of different grades given in percentage of graphite and clay by mass (1).**

| Pencil Grade | Graphite % | Clay % |
|---|---|---|
| 8B | 90 | 4 |
| 7B | 87 | 3 |
| 6B | 84 | 10 |
| 5B | 82 | 12 |
| 4B | 79 | 15 |
| 3B | 76 | 18 |
| 2B | 74 | 20 |
| B | 71 | 23 |
| HB | 68 | 26 |
| F | 66 | 28 |
| H | 63 | 31 |
| 2H | 60 | 34 |
| 3H | 58 | 36 |
| 4H | 55 | 39 |
| 5H | 52 | 42 |
| 6H | 50 | 45 |
| 7H | 47 | 47 |
| 8H | 44 | 50 |
| 9H | 41 | 53 |

To assess the behaviour of the pencil tip when abraded by stochastic collisions, a forensic analysis of particles shed by their wear was conducted, via image analysis of a 1 $cm^2$ patch of the porcelain plate atop the vibrating stage in our experimental set up, shown in Fig. 2A. The distribution of particle sizes, shown in Fig. S4, follows typical power-law statistics, with $\alpha \approx 1.8$. The long-tailed distribution shows that material removal proceeds predominantly through the scale-free shedding of minute submillimetric particles (that lack a characteristic particle size). Such distributions, with $1 < \alpha < 2$, are typical of wear processes, and are standard in the geomorphology literature on erosion, abrasion of rocks, etc. Therefore, the abrasion of pencil cores by random collisions in our experiments can reliably be considered a conventional example of mechanical wear rather than a material-specific or idiosyncratic process: the pencil tip is an archetypal biostinger analogue with unexceptional isotropic mechanical properties.

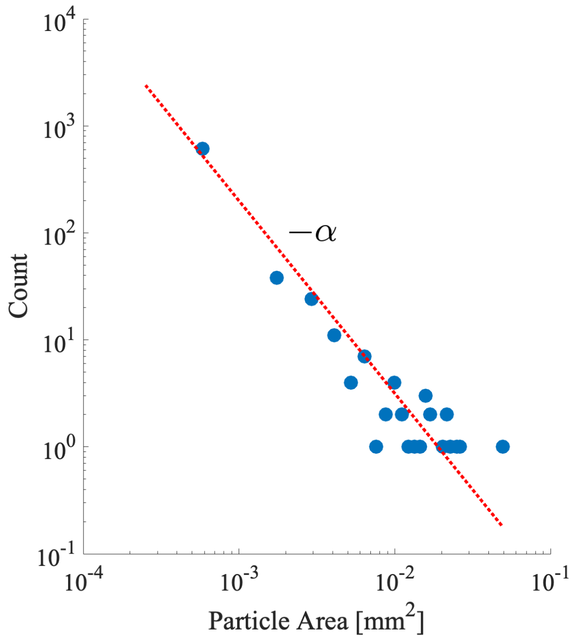


**Fig. S4. Distribution of size of particles shed by pencil stingers in the experiment. It follows typical power-law statistics with $\alpha \approx 1.8$, characteristic of scale-free, abrasive wear.**

## 3. Experiments using a "biomaterial pencil"

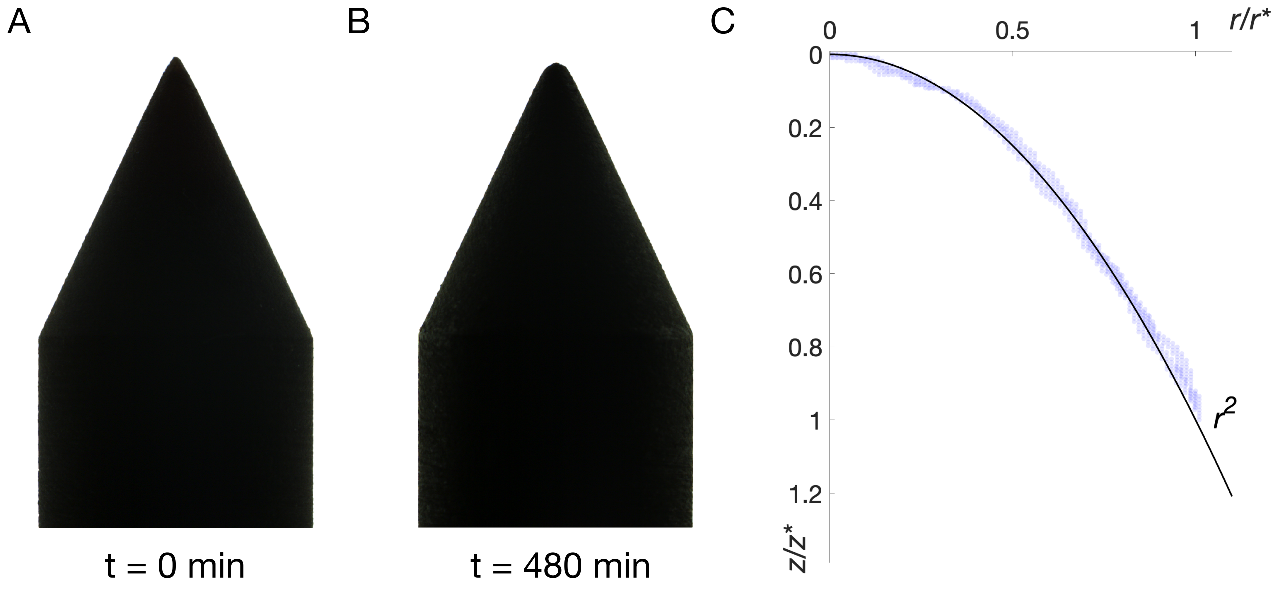


**Fig. S5. The shape evolution of a pencil-analogue carved out of the horn material of *B. taurus* (bull).(A) Initial profile of the bull horn sample. (B) Tip profile after 480 min of wear induced via stochastic collisions. (C) The extracted tip geometry follows $z \sim r^2$, in agreement with pencil experiments, and in alignment with the observed universal shape of biostingers.**

Experiments were conducted using a natural biostinger, the bull horn (from *B. taurus*). In tune with our pencil experiments, a laboratory scale specimen was machined out of the bull horn, forming a pencil-analogue made of this typical biostinger material, the details of which are furnished in Materials and Methods. Starting with its initial shape shown in Fig. S5A, the stinger is exposed to 480 minutes of stochastic collisional activity with arbitrarily shaped slices of the same material and similar dimensions, and wood encased graphite pencils. The resulting shape is given in Fig. S5B. Unsurprisingly, the worn tip exhibits the ‘universal’ parabolic profile as shown in Fig. S5C.

It is worth noting that unlike pencils, the entirety of the bull horn stinger and all its exposed surfaces are of the same material. Nonetheless, as expected from our model which follows the mechanistic view that wear rate is proportional to the local mean curvature, after exposure to collisional activity of $\sim 8$ hours, a relatively short span given the superior mechanical properties of the biomaterial compared to pencil cores, only the tip has undergone wear.

## 4. Supporting information for Figure 1

Dimensional values of $r^*, z^*$ used to normalise individual biostinger tip profiles in Figure 1A are given in Table S2. Also, the corresponding mean values for 7B and 2B pencils, used in Fig. 3B are indicated. Note that in addition to differences in material properties, the core radii of 7B and 2B pencils also differ, as mentioned in Table 1 in Materials and Methods.

**Table S2. Dimensional values of $r^*, z^*$ for the data set from Quan et al., PNAS (2024) (4), plotted in Fig 1A.**

| Biostinger | $r^*$ [$\mu m$] | $z^*$ [$\mu m$] |
|---|---|---|
| Radiolaria | 5.23 | 42.82 |
| Bee | 3.9 | 18.69 |
| Porcupine quill | 83.33 | 310.22 |
| Cactus | 335.88 | 4202.05 |
| Narwhal tusk | 13.46 x $10^3$ | 340.77 x $10^3$ |
| Hedgehog | 38.61 | 487.22 |
| Robinia | 1.22 x $10^3$ | 30 x $10^3$ |
| Scorpion | 46.3 | 167.3 |
| Whistling thorn | 850 | 29.88 x $10^3$ |
| 7B | 915 | 1320 |
| 2B | 665 | 850 |

Counterexamples of biostingers that deviate from the widely observed parabolic tip profile, are found mainly in pristine samples - stingers prior to deployment or usage. In addition to the examples shown in Figure 1B,C, an image of the jaw of a nurse shark (*G. cirratum*) displaying its multiple layers of teeth is reproduced from Ref. (5) in Fig. S6. In its natural state, inner (upper) rows of teeth are protected from exposure by epithelial tissue. As the outer (bottom) exposed row wears out, the teeth are shed, and the next row is exposed for use. The arrow in the Fig. S6 therefore represents age or time.

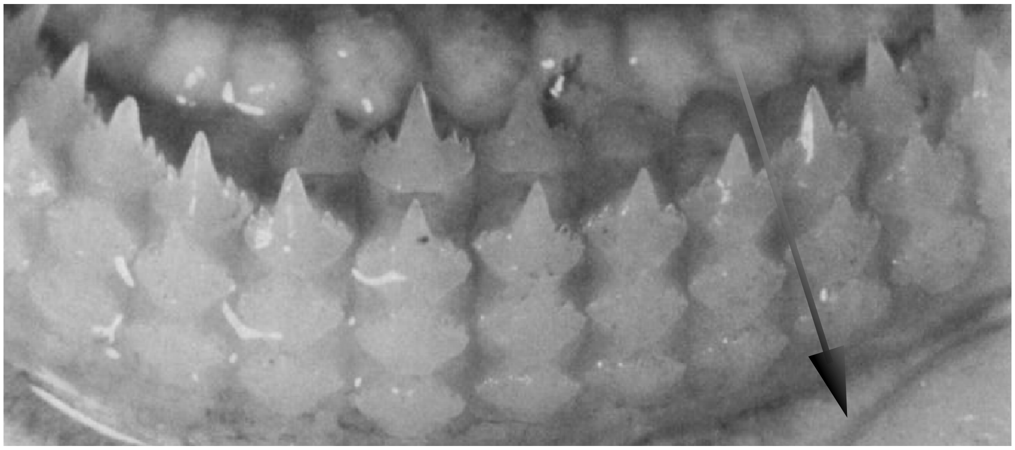

**Fig. S6. Effect of wear on rows of shark teeth: Outer rows show substantial wear, while the internal rows, protected underneath an epithelial cover, retain pristine teeth forms. The arrow, therefore, represents 'age' or time.**

## 5. Tip curvature

The exfoliatory shaping of the axisymmetric stinger by stochastic mechanical interactions proceeds in a self similar manner raising the likelihood that it will exhibit a parabolic tip profile ($n \approx 2$) at any arbitrary point in its lifetime. This is in spite of its instantaneous tip curvature, $\kappa_0(t)$, which represents the stinger's relative sharpness. It follows from our model that $\kappa_0(t) = \kappa_{00}/\sqrt{1 + 4D\kappa_{00}^2 t}$, where $\kappa_{00} = \kappa_0(0)$, from Equation 9 in Materials and Methods. Starting by representing the profile evolution of a 7B pencil from our experiments in terms of its local curvature $\kappa(r, t)$, we see that, as expected, the maximal curvature is at the axisymmetric centre or the instantaneous tip of the pencil, given in Fig. S7A. Since the tip profile is fragmented and not smooth at early intermediate time steps, these geometries are approximated using an interpolant polynomial of degree 10 for the current analysis. The progression of measured tip curvature is not inconsistent with the expression produced above as illustrated in Fig.S7B. Over long times, the expression simplifies asymptotically, at large $t$, to $\kappa_0(t) \simeq \sqrt{4Dt}$ as indicated in Fig. S7C. This suggests a convenient method to estimate $D$, the material and process dependent diffusivity, for biostingers in their natural environments.

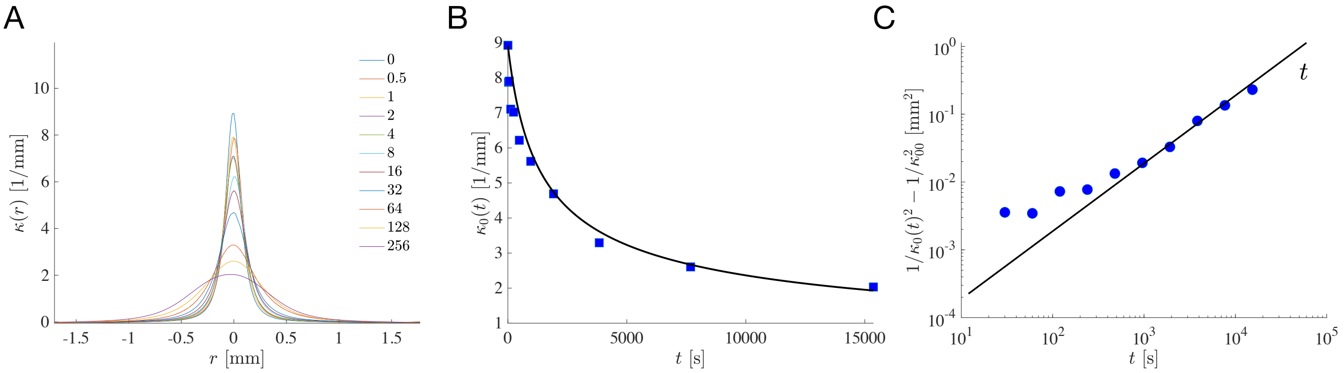


**Fig. S7. A representative example of the temporal evolution of tip curvature for a 7B pencil.(A) Tip profile is plotted in terms of its local curvature, $\kappa(r)$ for different time steps in the course of wear-driven morphing. (B) The relative sharpness of the stinger, represented by its tip curvature diminishes over time, in agreement with Equation 9 (see Materials and Methods). (C) At long times, the radius of tip curvature $1/\kappa_0$ scales as $\sqrt{4Dt}$ at long times, presenting a convenient method to estimate values of $D$.**

## Data Availability

Data in Figs. 1 to 3 available at: https://github.com/Jensen-Lab/sebastian2026.git

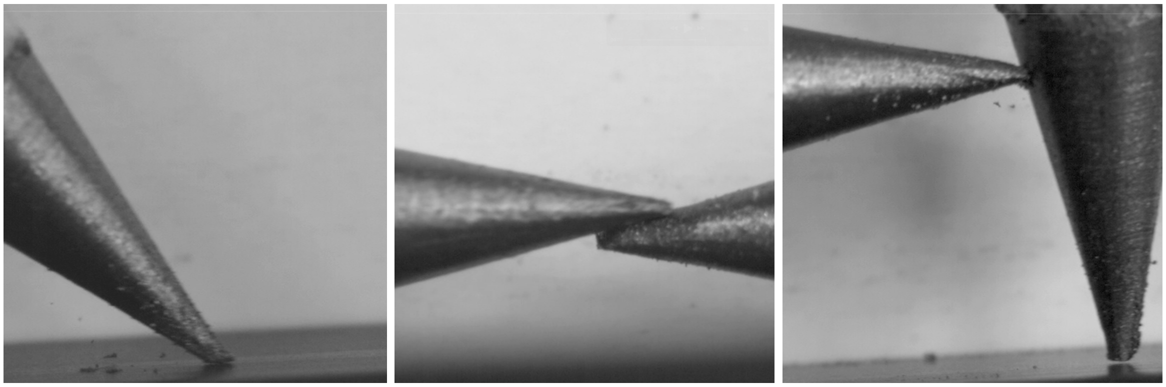

**Movie S1. Video sequence illustrating material removal during pencil-pencil collisions.**

## References


1. MC Sousa, JW Buchanan, Observational Models of Graphite Pencil Materials. *Comput. Graph. Forum* **19**, 27–49 (2000).
2. Faber-Castell, How we make pencils (2018).
3. H Petroski, *The Pencil: A History of Design and Circumstance.* (Knopf Doubleday Publishing Group, Westminster), (2011).
4. H Quan, et al., The shape of Nature's stingers revealed. *Proc. Natl. Acad. Sci.* **121**, e2316320121 (2024).
5. CA Luer, PC Blum, PW Gilbert, Rate of Tooth Replacement in the Nurse Shark, Ginglymostoma cirratum. *Copeia* **1990**, 182 (1990).
